# P-736. No Air Don’t Care: Lack of gas on imaging is associated with a longer time to surgical intervention in patients with necrotizing fasciitis

**DOI:** 10.1093/ofid/ofae631.932

**Published:** 2025-01-29

**Authors:** Amanda Stevens, Aveen Salar, America Silva, Alex Huang, Lauren Touleyrou, Sorabh Dhar, Lea Monday

**Affiliations:** Wayne State University School of Medicine, Franklin, Michigan; Wayne State University School of medicine, Detroit, Michigan; Wayne State University School of medicine, Detroit, Michigan; Detroit Medical Center, Detroit, Michigan; Wayne State University, Detroit, Michigan; Wayne State University/Detroit Medical Center, John Dingell VAMC, Detroit, Michigan; Wayne State University School of medicine, Detroit, Michigan

## Abstract

**Background:**

Although necrotizing fasciitis is a life-threatening infection, initial symptoms may be non-specific. The presence of gas on plain X-ray or computed tomography scan can be diagnostic, but is missing in up to 1/4 of cases.

**Figure 1.**
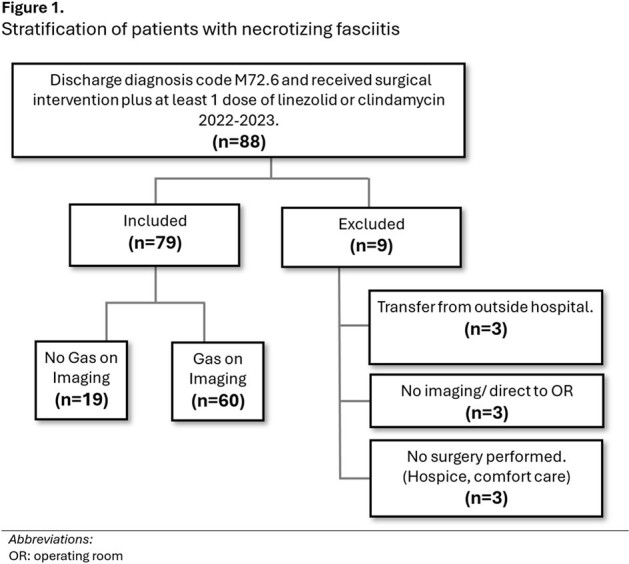
Stratification of patients with necrotizing fasciitis

**Methods:**

We performed a retrospective cohort study of adult patients admitted in 2022-2023 with necrotizing fasciitis (diagnosis code M72.6) at an urban level 1 trauma center, who received surgical intervention, and at least 1 dose of linezolid or clindamycin in addition to other antibiotics. Patients transferred from an outside hospital, who had no imaging results, or no surgical intervention were excluded. Patients with and without gas on initial imaging result were compared (Fig1). Primary outcome was whether a lack of gas on imaging correlated with longer time to surgical intervention. Time 1 was defined as the time radiographic images were attested, or the time of surgery consult (whichever was later). Time 2 was the documented operating room (OR) surgical start time. Clinical and microbiologic outcomes were also recorded.

**Table 1:**
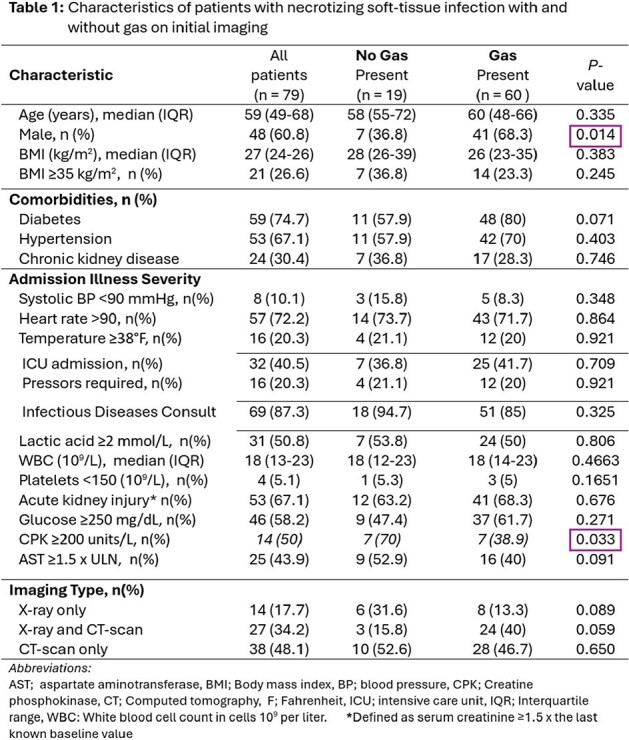
Characteristics of patients with necrotizing soft-tissue infection with and without gas on initial imaging

**Results:**

79 patients were included in the analysis (60 and 19 patients with and without gas on imaging, respectively). Cohorts were evenly matched in terms of illness severity and comorbidities, except that patients without gas on imaging were more often female (P=0.014) with higher rates of CPK elevation (P=0.033) (Table 1). Site of infection did not differ between groups (Fig2), nor did microbiologic OR culture results (figure not included, P=0.105). Patients without gas on imaging had significantly longer time to OR compared to those with gas (median 15.7 verses 4.3 hours, respectively, P=0.0128) (Figure 3a). Length of stay, repeat trips to OR, and mortality did not differ between groups, however, patients without gas had high rates of readmission (P=0.041) (Fig3b).

**Figure:**
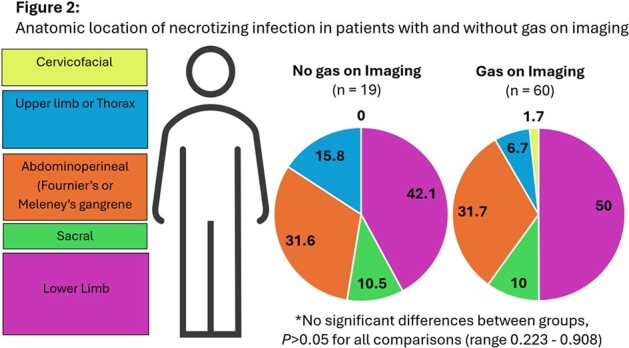

**Conclusion:**

Almost a quarter of necrotizing fasciitis patients presented without gas on initial imaging result. Despite having a similar degree of illness and poor prognostic laboratory parameters, these patients had a significantly longer time to OR compared to those with gas on initial imaging. Infectious diseases physicians should strive to provide valuable insight to our surgical colleagues for a syndromic approach to this illness regardless of the imaging results.

**Figure:**
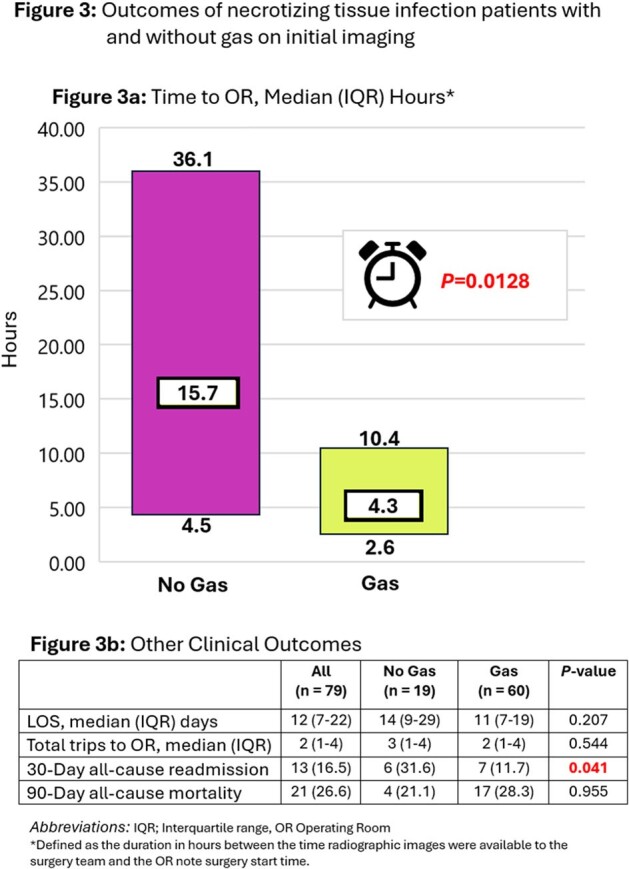

**Disclosures:**

**All Authors**: No reported disclosures

